# Nursing care for the transgender population in primary health care: an integrative review*

**DOI:** 10.17533/udea.iee.v41n1e07

**Published:** 2023-03-14

**Authors:** Manoella Alves Carneiro Chagas, Adriano Maia dos Santos, Naila Neves de Jesus

**Affiliations:** 1 Nurse, Resident,. Instituto de Medicina Integral Professor Fernando Figueira, Recife (Brazil). Email: manuacc28@gmail.com Instituto de Medicina Integral Professor Fernando Figueira Instituto de Medicina Integral Professor Fernando Figueira Recife Brazil manuacc28@gmail.com; 2 Dentist, Doctor. Associate Professor, Universidade Federal da Bahia, Vitória da Conquista (Brazil). Email: maiaufba@ufba.br Universidade Federal da Bahia Universidade Federal da Bahia Vitória da Conquista Brazil maiaufba@ufba.br; 3 Pharmacist, Doctor. Universidade Estadual do Sudoeste da Bahia, Vitória da Conquista (Brazil). Email: naila.neves@yahoo.com.br Universidade Estadual do Sudoeste da Bahia Universidade Estadual do Sudoeste da Bahia Vitória da Conquista Brazil naila.neves@yahoo.com.br

**Keywords:** transgender persons, gender identity, nursing care, primary health care, personas transgénero, identidad de género, atención de enfermería, atención primaria de salud, pessoas transgênero, identidade de gênero, cuidados de enfermagem, atenção primária à saúde

## Abstract

**Objective.:**

To describe the care provided to the transgender population by nursing in Primary Health Care (PHC).

**Methods.:**

Integrative literature review performed in the Virtual Health Library (VHL), Medline/PubMed and Web of Science (WoS) databases without a pre-established time frame, using the descriptors “transgender persons”, “gender identity”, “nursing care” and “primary health care”.

**Results.:**

Eleven articles published between 2008-2021 were included. They were categorized as follows: Embracement and healthcare; Implementation of Public Health Policies; Weaknesses in academic training; Barriers between theory and practice. The articles showed a limited scenario of nursing care for the transgender population. The scarcity of research focused on this theme is an important sign of how care has been incipient or even non-existent in the context of PHC.

**Conclusion.:**

Structural and interpersonal stigmas materialized in discriminatory and prejudiced practices perpetrated by managers, professionals and health institutions constitute the greatest challenges to be overcome for comprehensive, equitable and humanized care provided to the transgender population by nursing.

## Resumen

**Objetivo.:**

Describir los cuidados de enfermería en atención primaria de salud brindados a la población transexual.

**Métodos.:**

Revisión integrativa realizada en las bases de datos de la Biblioteca Virtual en Salud (BVS), Medline/PubMed y Web of Science, sin marco temporal preestablecido y utilizando los descriptores “transgender persons”, “gender identity”, “nursing care” y “primary health care”.

**Resultados.:**

Se incluyeron 11 artículos, publicados entre 2008 y 2021, categorizados así: Recepción y asistencia sanitaria; Aplicación de políticas de salud pública; debilidades en la formación académica; Barreras entre la teoría y la práctica. Los artículos analizados en esta revisión mostraron un panorama limitado de la forma en que los cuidados de enfermería se han brindado a esta población. A pesar de la escasez de investigaciones sobre este tema la revisión mostró que la atención ha sido incipiente o incluso inexistente en el contexto de la Atención Primaria en Salud.

**Conclusión.:**

El estigma estructural e interpersonal, materializado en prácticas discriminatorias y preconcebidas realizadas por gestores, profesionales e instituciones de salud, constituye el mayor desafío a superar para una atención integral, equitativa y humanizada a la población transexual por parte de la enfermería.

## Resumo

**Objetivo.:**

Descrever a assistência prestada pela enfermagem à população transgênero na Atenção Primária à Saúde (APS).

**Métodos.:**

Revisão integrativa da literatura, realizada nas bases de dados da Biblioteca Virtual em Saúde (BVS), Medline/PubMed e Web of Science (WoS), sem recorte temporal pré-estabelecido e utilizando-se dos descritores ““transgender persons”, “gender identity”, “nursing care” e “primary health care”.

**Resultados.:**

Foram incluídos 11 artigos, publicados entre 2008-2021, assim categorizados: Acolhimento e cuidados em saúde; Implementação de Políticas Públicas de Saúde; Fragilidades da formação acadêmica; Entraves entre a teoria e a prática. Os artigos mostraram um panorama limitado sobre a assistência de enfermagem à população transgênero. A escassez de pesquisas voltadas para esta temática configura-se como um ponto importante de como o cuidado tem sido incipiente ou mesmo inexistente no contexto da APS.

**Conclusão.:**

O estigma estrutural e interpessoal, materializados em práticas discriminatórias e preconceituosas perpetradas por gestores, profissionais e instituições de saúde, constituem os maiores desafios a serem superado para uma atenção integral, equânime e humanizada à população transgênero pela enfermagem.

## Introduction

In Brazil, health is a constitutional right guaranteed since 1988, from the creation of the Unified Health System (SUS), regulated by Federal Law 8080/90, which consolidates health as “a fundamental human right, and the State must provide the indispensable conditions for its full exercise”.^1^ The basic principles of the SUS are universality, integrality and equity. The latter aims to reduce social inequalities, which means recognizing the existence of different social groups that require specific and differentiated care to meet their needs and overcome social injustices.^2^^,^^3^ In 2011, the National Policy for the Comprehensive Health of Lesbians, Gays, Bisexuals, Transvestites and Transsexuals (Portuguese acronym: PNSI-LGBT),^4^ was established as a response to the demands and specificities of this population, which were made invisible. The institution of the PNSI-LGBT represents a historic milestone of resistance and struggle of this population to guarantee their rights.^5^ Prior to the creation of this policy, the launch of the Brazil Without Homophobia Program in 2004,^6^ also fruit of the coordination between civil society and the State, and a milestone for the strengthening of citizenship, rights and dignity for the LGBTQIA+ population also stands out. The access to health services free of any discrimination is one of the main agendas of this community.^3^^,^^7^

Discrimination based on sexual orientation and gender identity is characterized as a social determinant of health as it triggers processes of suffering, illness and premature death resulting from different types of violence perpetrated against the LGBTQIA+ population.^8^^,^^9^ In addition, formal access to health services is permeated by symbolic, technical or organizational barriers that commonly depersonalize care and produce inequities.^10^ The transgender population faces greater difficulties in social insertion because their differences are marked in their bodies, often read as abject and, thus, placed on the social margins.^10^ Transgender people do not identify with the gender assigned to them at birth, and may identify as a transsexual man, transsexual woman or transvestite, whether or not they are in the man or woman binarity.^11^ In this scenario, transgender people have specific health needs, such as carrying out body modifications through hormone therapy, gender reassignment surgery, among others, often demanded for their gender affirmation. Thus, Primary Health Care (PHC), as the main and priority door of the Health Care Network (Portuguese acronym: RAS),^12^ and nurses as components of the minimum team in basic health units, play a fundamental role in this process, providing general and specific care and acting in user embracement for the production of comprehensive care.^13^^,^^14^

According to the National Primary Care Policy of 2017,^15^ nurses are important professionals for this articulation, as they act directly in the management, planning and care in the different spaces of PHC.^14^ Therefore, nursing professionals in PHC must know the community and establish bonds, perform and supervise user embracement with qualified listening and risk classification according to established protocols, promote disease prevention and health protection actions, identify individual and collective health problems, implement and keep routines, protocols and flows related to their area of competence at the basic health unit updated, work with groups by respecting and interacting with cultural differences, demonstrate knowledge of the population’s health problems and of social determinants, among others.^15^^,^^16^ In view of this, nurses in PHC, as members of the team, have a fundamental role in the construction of spaces for embracement of the transgender population, performing functions based on the principles of the SUS, not admitting discrimination of any kind.^17^

In this context, in spite of what is provided for by legislation and health policies, care to the transgender population has been neglected by PHC professionals.^10^ This population faces different types of barriers to access health services, such as weaknesses in user embracement, inadequate training of professionals and incipient implementation of the PNSI-LGBT.^5^ These barriers also result from social prejudice and stigma, which act through institutional discrimination and distance transgender patients from health services.^17^ Therefore, the aim of this review of the literature is to describe the care provided to the transgender population in PHC by nursing.

## Methods

This is an integrative literature review, a method that provides the synthesis of knowledge and incorporation of the applicability in practice of results from significant studies.^18^ It was carried out through six steps: 1) choice of theme and elaboration of the guiding question; 2) literature search or sampling; 3) data collection; 4) critical analysis of included studies; 5) discussion of results; and 6) presentation of the integrative review. The Preferred Reporting Items for Systematic Reviews and Meta-Analyses (PRISMA) statement was adopted as a guideline for its development.^19^

In order to answer the guiding question of the study: “How has nursing care been provided to the transgender population in PHC?”, in December 2021, a search was performed in databases of the Virtual Health Library (VHL), Public Medline (PubMed) and Web of Science (WoS) using the descriptors “*Pessoas Transgênero”, “Identidade de gênero”, “Cuidados de Enfermagem*” and “*Atenção Primária à Saúde*”, from the Descriptors in Health Sciences (DeCS) and its correspondents in English “Transgender persons”, “Gender identity”, “Nursing care” and “Primary health care”, from Medical Subject Headings (MeSH). Terms in both languages were combined using the Boolean operator OR for the first two terms, and AND for the other terms.

Regarding selection criteria for publications, complete articles in Portuguese, English and Spanish available electronically, portraying the theme related to the review were included. Duplicated articles and those not directly addressing the theme were excluded. A timeframe was not established for the searches in order to increase the scope of the research.

**Figure 1:**
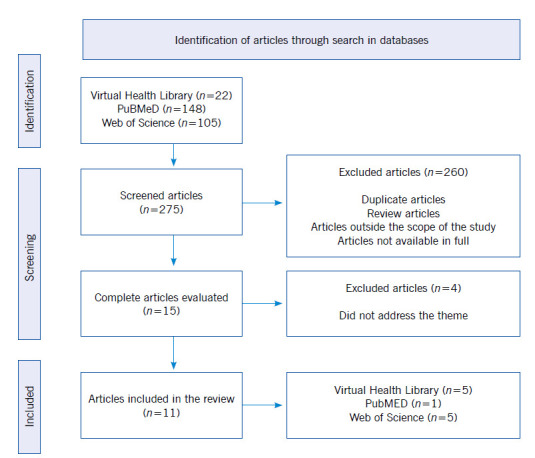
Publication selection flowchart

At first, 22 articles were found in VHL, 148 articles in PubMed and 105 articles in WoS, totaling 275 articles. The analysis performed by reading the records led to the selection of 11 articles (Figure 1).

After selecting the articles, a spreadsheet containing the title, author, country of origin, year of publication, journal title, objectives, main results and conclusions was created in Excel 2010. It constituted the study database. The articles were grouped, producing categories that were presented, analyzed and discussed in light of the available literature on the subject.

## Results

The 11 studies analyzed were published between 2008 and 2021 in ten different journals. With regard to the countries of origin of publications, five were from Brazil,^20^^-^^24^ two from Canada^25^^,^^26^ and four from the United States.^27^^-^^30^ Five articles were published in Portuguese and six in English. Articles in Spanish were not selected. The general characteristics of included studies are systematized in Table 1.

**Table 1:** General characteristics of included studies

Reference 20. Oliveira EM, Oliveira JF, Suto CSS, Porcino C, Almeida SP, Oliveira DS. Institutional Health Spaces as “no place” of tranvestites in the social representations of nurses. Rev. Baiana Enferm. 2020; 34:e35603. Country: Brazil
Objective. To discuss the invisibility of the transvestite person in health institutions based on nurses’ social representations.
Type of study. Qualitative study using social representation.
Main findings. The invisibility of transvestites is implied in the way nurses perceive the need/possibility of occupying these spaces, especially those that offer primary care.
Conclusion. The social representations of investigated nurses revealed meanings for invisibility, exclusion, difficulties in service and care provision to transvestites in health institutions. The invisibility identified in nurses’ representations occurs because of the way health professionals perceive the need/possibility of occupying these spaces.
Reference 21. Oliveira EM, Oliveira JF, Porcino CA, Campos LCM, Reale MJOU, Souza MRR. “Male body with female gestures?”: a transvestite’s image made by nurses. Interface (Botucatu). 2019; 23:e170562. Country: Brazil
Objective. To describe the image of the transvestite person revealed by nurses.
Type of study. Qualitative study using the Free Word Association Test (FWAT).
Main findings. The investigated group revealed an image about the transvestite based on elements of the biological and social dimensions, sexual orientation and gender identity; and points to the emergence of a progressive perspective on the way of being a transvestite.
Conclusion. Reflecting on the heterogeneous, deep and broad demands involving transvestitism will help nurses in their various areas of activity: to rethink about individualized modes of care, develop care protocols, train professionals and contribute in order to raise awareness of the team regarding respect for differences and human rights.
Reference 22. Sehnem GD, Rodrigues RL, Lipinski JM, Vasquez MED, Schmidt A. Technical-scientific (un)prepare for transvestites care: nurses’ perceptions. Rev. Enferm. UFSM. 2017; 7(2):236-47. Country: Brazil
Objective. To know nurses’ perceptions of transvestites and the technical-scientific preparation to assist them.
Type of study. Descriptive qualitative study.
Main findings. Nurses are unaware of the meaning of being a transvestite, limiting it to the figure of a man dressed as a woman. Important gaps were perceived in the academic training of these professionals regarding the specificities of care for transvestites. With regard to professional practice, the lack of knowledge about public policies and legislation aimed at this population was evident.
Conclusion. The study showed that healthcare actions aimed at the care for transvestites are not developed. The few initiatives presented occurred in an isolated and fragmented way, based on individual initiatives by some nurses.
Reference 23. Sehnem GD, Rodrigues RL, Lipinski JM, Vasquez MED, Schmidt A. Health care assistance in primary care: access to care. Rev. Enferm. UFPE. 2017; 11(4):1676-84. Country: Brazil
Objective. To know healthcare for transvestites from the perspective of nurses in primary care.
Type of study. Descriptive qualitative study.
Main findings. Primary healthcare was not the gateway to healthcare for transvestites, as the service is not structured to serve this population. User embracement was presented as a tool for the implementation of care for transvestites.
Conclusion. The study showed that healthcare actions aimed at the care for transvestites are not developed. The few initiatives presented occurred in an isolated and fragmented way, based on individual initiatives by some nurses.
Reference 24. Santos JS, Silva RN, Ferreira MA. Health of the LGBTI+ population in Primary Health Care and the insertion of Nursing. Esc. Anna Nery Rev. Enferm. 2019 ;23(4):305-10. Country: Brazil
Objective. To reflect on approaches to the health of the LGBTI+ population, Primary Health Care and nursing in the care of this population.
Type of study. Essay.
Main findings. Nurses in Family Health teams must know the main demands of the LGBTI+ population. The institutional reorientation of PHC poses new challenges to the realization of the right to health for the LGBTI+ population.
Conclusion. Considering that the LGBTI+ population is also under the responsibility of Nursing within the scope of PHC, the provision of comprehensive care with a view to minimizing the inequalities suffered by this population is also responsibility of this professional category.
Reference 25. Ziegler E. The integral role of nurses in primary care for transgender people: a qualitative descriptive study. J. Nurs. Manag. 2020; 29(1):95-103. Country: Canada
Objective. To explore the specific nursing activities for provision of primary care for transgender individuals and assess which methods have supported the development of their competence in providing primary care for transgender individuals.
Type of study. Descriptive qualitative study.
Main findings. Nurses are important in primary care for transgender people. One of the main challenges was the lack of education, but mentoring and collaboration contributed to skills development. Ensuring the workplace provided gender-affirming care was key to a safe and inclusive environment.
Conclusion. Supporting nurses to build capacity and work across the scope of practice can improve access to care. Ongoing mentoring opportunities and ensuring an inclusive workplace will help provide care for this vulnerable population.
Reference 26. Ziegler E, Valaitis R, Risdon C, Carter N, Yost J. Models of care and team activities in the delivery of transgender Primary Care: an Ontario case study. Transgend. Health. 2020 ;8(2):122-8. Country: Canada
Objective. To explore how primary care for transgender individuals is provided within different models of primary care in Ontario and the roles that primary care team members play in the provision of care, barriers, enablers, as well as the clinical competence of professionals in providing transgender care.
Type of study. Case study. Qualitative research.
Main findings. Professionals were prone to working independently. In cases with an interdisciplinary team, collaboration was limited. Nurses, physicians and counselors contributed the most to care. The main challenges included the lack of coordination of services within organizations and the need for professional education.
Conclusion. Primary care professionals who provide care to transgender individuals are working hard to improve access to care and remove barriers. Providing primary care services to transgender people is within the scope of practice of primary care professionals and can be part of routine care. This study identifies areas to further improve care delivery. The results support the development of educational initiatives that include providing care for transgender people.
Reference 27. Dutton L, Koenig K, Fennie K. Gynecologic care of the female-to-male transgender man. J. Midwifery Womens Health. 2008; 53(4):331-7. Country: United States
Objective. To inform health professionals of the gynecological needs of the male transsexual community.
Type of study. Qualitative study and use of the Health Care Relationship Trust Scale.
Main findings. Four themes were prominent regarding transgender men’s experiences: 1) receiving annual gynecological care was perceived as important; 2) breasts caused the greatest gender identity conflict; 3) disclosing gender identity to health professionals was a tense moment; and 4) the “male/female” binomial in medical records and the use of pronouns by the medical team were barriers to healthcare.
Conclusion. Assessing the gynecological care needs of transgender men helps to characterize the barriers faced by them when seeking healthcare.
Reference 28. Markwick L. Male, Female, Other: Transgender and the Impact in Primary Care. J. Nurse Pract. 2016; 12(5):330-8. Country: United States
Objective. To Inform nurses about the health needs of the transgender person, outline the basic care and issues that may arise in a primary care setting.
Type of study. Literature review.
Main findings. The article reviews terms, concepts, demands/needs and clinical approaches to improve clinical care and the embracement of transgender people in primary care services. In addition, it brings reflections on the role of nursing in the care for the transgender population and on the inadequate university training of nursing to deal with this population.
Conclusion. There is a lack of appropriate education to conduct care for transgender people in nursing and medical schools, and lack of available resources to promote adequate clinical care, which implies a great disparity in terms of access to quality care by transgender people.
Reference 29. Singh SM, Gatzke N. The Nurse Practitioner‘s Role in the Management of Gender Dysphoria Among Youth. J. Nurse Pract. 2021; 17(5):540-4. Country: United States
Objective. To understand the different care possibilities of nurses to assist transgender people in PHC.
Type of study. Essay.
Main findings. Nurses can improve health outcomes and expand access to equitable healthcare for transgender people. Psychological assessments are mandatory to determine the extent of gender incongruence. Immediate care leads to better health outcomes and decreased suicide rates.
Conclusion. Nurses need to be well-versed in issues related to gender identity while providing culturally competent care. Nurses should have a thorough understanding of treatment options for gender dysphoria, including pubertal block and cross-sex hormone therapy.
Reference 30. Abeln B, Love R. Considerations for the Care of Transgender Individuals. Nurs. Clin. North Am. 2019; 54(4):551-9. Country: United States
Objective. To expose the need to understand the health demands of the transgender population and present culturally competent terminologies, surgeries and gender-affirming treatments available to transgender individuals.
Type of study. Essay.
Main findings. An understanding of culturally competent transgender terminology better prepares nurses to care for this population and reduces transgender-related discrimination in the healthcare setting. Furthermore, when health professionals are aware of gender-affirming surgeries and treatments available to transgender individuals, they are better equipped to provide care and refer them to specialists. Improvements in the care of transgender individuals in the healthcare setting will eventually fill the gap in health disparities seen in this population.
Conclusion. It is critical that improvements are made to the healthcare environment for better outcomes and to better prepare healthcare professionals to care for the transgender population.

After reading and structuring the information from selected studies, four thematic categories emerged: I - Embracement and healthcare; II - Implementation of Public Health Policies; III - Weaknesses in academic training; IV - Barriers between theory and practice.

### Category I - Embracement and healthcare

Studies have demonstrated the rarity of the presence of transgender people in health services, showing the disrespect for a fundamental human right.^20^^,^^22^^-,^^24^^,^^27^^,^^28^ This results mainly from the way they are treated, with moral judgment and the abjection of their bodies, denounced by the gestures, looks and speeches of professionals who assist them in health services.^20^ In Brazil, the care provided to this population in the PHC by nursing is focused on the delivery of condoms and HIV/AIDS testing. When other approaches are needed, the care offered by health professionals becomes permeated by doubts, difficulties and inconsistencies.^23^ Even so, nurses report that they either have never assisted or assisted an incipient number of people who recognized themselves as transgender, which demonstrates the negligence suffered by this group and the urgency of implementing strategic actions and investments aimed at a PHC that accepts and takes care of the entire population in a universal, equitable and comprehensive way.^20^^-,^^24^

In this context, user embracement is the main strategy used to bring this population closer to health services and to enhance the implementation of their assistance in PHC. Some studies timidly address how the embracement of transgender people has been carried out and point out ways to structure a PHC prepared to meet their needs.^25^^,^^26^ On the other hand, other studies demonstrate the lack of access together with the technical-scientific unpreparedness of nursing professionals, in addition to the stigma, including to offer user embracement.^23^^,^^28^ Nurses report not feeling qualified, despite recognizing its importance. This technical disqualification is reflected in the lack of access to health services for individuals who do not follow the normative binary standard, which ends up transforming a place that should be recognized as space of user embracement and care into a “non-place”.^20^

Regarding strategies adopted in user embracement, the articles show the use of the pronoun by which the person wants to be called, respect for the social name, the display of signs of a safe space (use of non-binary forms in the environment), neutral bathrooms in terms of gender issues and safe space training for all employees. These changes in ambience and the investment in the training of all professionals, especially those acting in user embracement, have been configured as effective actions to bring the transgender population closer to PHC services.^25^ With regard to the role of health professionals, the articles briefly describe the care provided in PHC: care related to gender transition, acute episodic problems and management of chronic diseases.^25^^,^^26^ As for the activities performed by nursing, they highlight the general and specific care provided to the transgender population, such as: management of chronic diseases, counseling, diagnostic testing, episodic (acute) care, health assessment, health promotion, health education (guidance on medication, administration of injectable hormones, etc.), prescription of medication and preventive care.^24^^,^^25^

During user embracement, nurses must provide qualified listening, identifying the user’s health needs. The anamnesis must be detailed and expanded, paying attention to issues permeating the transgender population, collecting personal history, health and family history, current use of medications, immunizations, etc. At this point, it is relevant to consider the history of violence, which is very present in the daily life of these patients,^20^^,^^21^ providing necessary guidelines and referrals. Guidance should also be provided on possibilities of care, especially with regard to body changes (transsexualization process, etc.), if this is the demand.^24^^,^^30^

### Category II - Implementation of Public Health Policies

In Brazil, the implementation of the PNSI-LGBT is one of the biggest challenges with regard to improving healthcare for the transgender population. More than a decade has passed since its publication, and health of this group is still marked by lack of access and the curtailment of the right to health.^23^

This fact shows the relevance of the management component in this process of legitimizing care practices. Although some studies^25^^,^^26^ bring extremely positive experiences, the need for management support and the development/implementation of public policies is an important critical issue. According to the study analyzed,^26^ nurses were not aware of any specific organizational policy for the transgender population in their workplaces. Organizational policies are a set of rules or principles that serve as a resource for employees, facilitate adherence to standards of practice, and influence decision-making and activities within the health service. Its absence makes it difficult to systematize care directed at the group under study. A Canadian study^26^ shows that despite the lack of policies to accommodate transgender individuals, the implementation of actions aimed at this public in PHC is already demonstrated in some health units that have become a reference in the territory, with ongoing training made available to all health professionals, team meetings to discuss the cases and weekly meetings via teleconference mediated by specialist professionals. These actions corroborate the development of treatment plans/care flows and, consequently, improvements in healthcare. The same study points out the ‘Guidelines and protocols for hormone therapy and primary healthcare for trans clients’ as the main reference used in practice. All these activities, including the protocol, are carried out by the Rainbow Health Ontario, which is a Sherbourne Health program designed to improve access to services and promote the health of the LGBT community. It also receives funding from the Ministry of Health to act as a catalyst for improving services, increasing knowledge, presenting innovative practices and encouraging networking and collaboration, highlighting the importance of social participation in building advances in favor of the transgender population.^26^

### Category III - Weaknesses in academic training

Weakness in academic training was identified in all analyzed studies. Nurses’ lack of knowledge about the demands of the transgender population makes the identification of the health needs of this population invisible, and neglects care within the scope of PHC.^22^^,^^28^ In addition to academic training, this fragility is also attributed to the absence of institutional spaces for ongoing education in the work process that discusses healthcare for transgender people.

Misinformation and moral values can be overcome through the development of cultural competence to deal with the specificities of the transgender population within the scope of PHC. Unfortunately, in nursing and medical schools, there is still a lack of adequate education regarding the care of transgender people, and a lack of available resources to promote adequate clinical care, causing great disparity in terms of access to quality care for the transgender person.^28^

### Category IV - Barriers between theory and practice

Among the 11 articles analyzed, only three^23^^,^^25^^,^^26^ actually address nursing care experiences in PHC. The other articles, in general, deal with theoretical aspects of care contained in policies through reviews and critical essays related to the lack of access and the incipient training of professionals.^24^^,^^28^^-^^30^ Such findings indicate the need to move beyond theory, using it to improve the care practice in different areas of healthcare. The difficulty of the transgender population in access to PHC services is a consensus in all articles, and they all refer to strategies to bring the transgender population closer to health services and focus on training and information for the qualification of nursing practices in PHC. In this scenario, the stigma, prejudice and discrimination experienced by the transgender population in health services^22^ were the tonic to be overcome, in addition to the still hegemonic biomedical model to the detriment of user embracement.

## Discussion

Publications converge on the need for user embracement services that respect the use of the social name, the pronoun by which the individual wishes to be identified, display of safe space signs (use of non-binary forms), gender-neutral bathrooms and training with cultural competence for all professionals. Fragilities in user embracement, academic training, insufficient in-service education for professionals and negligence in the implementation of public policies on transgenderism indicate important obstacles in accessing PHC health services.

In Brazil, nursing care aimed at transgender individuals in PHC is still incipient and restricted to dispensing condoms and testing for HIV/AIDS. On the other hand, studies conducted in Canada indicate ways to structure a PHC prepared to meet the needs of this group, highlighting how nursing care has been practiced in these spaces.^25^^,^^26^ Furthermore, there is a need to create care protocols that support and provide subsidies to nursing for an adequate practice in the care of the transgender population. To this end, it is suggested that nursing^24^^,^^28^^,^^29^ acting in PHC demands from class and manager councils in order to modify the reality of the health of the transgender population in their different territories.

User embracement is an operational guideline of the SUS, which has the following principles: to assist all people who seek health services, guaranteeing universal accessibility and assuming its main role of listening to the demands of the population, therefore being resolutive; to reorganize the work process aimed at its displacement from the central axis, which is the doctor, to a multidisciplinary team in charge of the care process, committing itself to solve users’ health problem; to qualify the worker-user relationship, which should be based on humanitarian, solidarity and citizenship parameters.^4^^,^^31^^,^^32^ In this sense, user embracement is a fundamental tool for an inclusive work process with the potential to break barriers of access in PHC, as it mobilizes the sensitivity of health workers, requiring reflective action, ethical and solidary development to listen and dialogue, generating reciprocal satisfaction.^13^

User embracement is still a major challenge for the SUS, especially when directed at the transgender population,^10^ who requires health professionals with knowledge about gender and sexuality, who break social taboos in a way to overcome many of the discriminations experienced by this public.^5^^,^^33^ Thus, it is necessary to break with social stigmas, address concepts permeating the transgender population and know their main demands and needs.^24^^,^^30^

It is important that professionals reflect on their practice, reflect on the population of their territory and their demands, and be transforming agents.^33^^,^^34^ In Brazil, for example, campaign models, such as “Pink October” and “Blue November” determine the gender to which these campaigns are aimed (women and cisgender men), making the transgender population completely invisible in this process, as well as their health needs, such as gynecological ones (for transgender men and people with vagina/uterus), urology (for transgender women and people with prostate) etc., reinforcing exclusionary practices.^10^^,^^27^ Thus, nursing has much to contribute to improve health access for the transgender population in PHC, making it possible, together with a multidisciplinary team, that Basic Health Units become places to which the transgender population feel they belong and where their health demands are met.^28^^,^^29^ In this context, the managers’ inertia in the face of the urgent need to implement the PNSI-LGBT and in the development of care strategies (care flows, protocols, among others), especially in the context of PHC, shows the marginalization of the demands of this population in the SUS, evidencing multiple barriers.^5^ All that also converges with the Report of the First National LGBT Health Seminar, which points to the lack of training of managers and health professionals as an important critical point in the implementation of the PNSI-LGBT.^35^

Nursing plays an extremely important role, among others, as a PHC component that can contribute to the care of the transgender population.^24^ The nursing professional must encourage and support the implementation of the PNSI-LGBT, keeping routines and protocols up to date, as well as care flows related to their area of competence at the Basic Health Unit.^15^ In addition, they can make proposals at PHC team meetings, stimulating changes in the ambience, user embracement and care flows. Furthermore, Nursing can discuss and develop strategies with the team to bring the Basic Health Unit closer to the transgender population in an active search movement in the territory. In this sense, the construction of health actions should also be performed together with this population, admitting their protagonist role in the production of their health.^7^^,^^36^

An Australian study found that the incipient knowledge and unpreparedness of professionals are the most important obstacles to the LGBT population’s access to health services.^33^ These limitations in academic training reinforce the cis-heteronormative character reproduced in educational institutions, in which the theme under study is addressed only on occasion in their curriculum matrices.^37^ In Brazil, the scarcity of discussions on the subject during undergraduate nursing courses is due to outdated curriculum matrices centered on traditional models that do not broadly include professional nursing education in curricular components addressing care with an approach to gender and sexuality.^38^ Many studies, not only in the national context, discuss the existing lack of integration in nursing courses of contents addressing care to the transgender population in a transversal manner.^28^^,^^39^ The limited approach to this theme in the academic training of nursing professionals has contributed to the inflexible care based on individual and collective discriminatory values, reduction of the demands of the transgender population to HIV/AIDS, thereby reinforcing stigmas and disregarding the health of this group in a broader sense.^32^

## Conclusion

The articles analyzed in this literature review showed a limited overview of nursing care for the transgender population, given the few studies found at the national and international level. Nonetheless, the scarcity of academic works focused on this theme is an important indication that care has been incipient or even non-existent in the context of PHC.

It was also evident that structural and interpersonal stigmas materialized in discriminatory and prejudiced practices perpetrated by managers, professionals and health institutions constitute the greatest challenges to be overcome for comprehensive, equitable and humanized care for the transgender population. Stigma, in turn, produces discrimination, which is materialized by social exclusion and the most varied forms of violence, including institutional violence, present in various social structures that directly affect transgender people’s access to health services. Given the above, there is an urgent need to reorganize the curricular matrices of nursing courses, so that they also address the health of the transgender population in a transversal way to the training process.

Study limitations. This is a review text, and the chosen databases may not reflect the set of studies, even though they adequately signal the productions related to nursing care for the transgender population. In addition, many successful practices may be hidden and are not properly disclosed, as publishable academic work often discloses empirical results that privilege practices with other social groups - structural stigma is also part of scientific production.
